# Epigallocatechin-3-Gallate Restores the Pulmonary Keap1-Nrf2-HO-1 Pathway and Attenuates Bile Acid-Associated Lung Injury in a Mouse Model of MASH

**DOI:** 10.3390/ijms27208948

**Published:** 2026-10-09

**Authors:** Ahmad Salhab, Johnny Amer, Rifaat Safadi

**Affiliations:** The Liver Institute, Gastroenterology Institute, Hadassah-Hebrew University Hospital, Jerusalem 91120, Israel

**Keywords:** liver–lung axis, bile acids, MASH, lung injury, EGCG

## Abstract

The crosstalk between the liver and the lung is complex and not yet fully understood; however, recent studies suggest that metabolic dysfunction, often originating in the liver, can influence lung injury and other lung diseases. The current study investigates the role of bile acids (BA) in mediating lung injury in a mouse model of metabolic dysfunction-associated steatohepatitis (MASH). *Ob*/*Ob* mice were fed a high-fat diet (HFD) for 4 weeks to establish a MASH model. Littermate mice were used as controls. Mice of each genotype were randomized to epigallocatechin-3-gallate (EGCG; 200 mg/kg/day, oral gavage) or vehicle. BA levels were assessed in serum and bronchoalveolar lavage fluid (BALF), and lung and liver tissues were obtained and evaluated for histological damage, epithelial apoptosis, and oxidative stress markers. MASH mice exhibited significant pulmonary injury, characterized by elevated pulmonary *Sftpd* mRNA expression (2.10 ± 0.68-fold) and sRAGE levels (986 ± 144 pg/mL), together with increased apoptosis of alveolar type I (30.0 ± 8.0%) and type II (32.4 ± 7.8%) epithelial cells, indicating impaired alveolar integrity. Pulmonary oxidative stress was evidenced by increased malondialdehyde (MDA) levels (0.67 ± 0.11 nmol/mg tissue) and a marked reduction in superoxide dismutase (SOD) protein levels (6.25 ± 3.30 ng/mg tissue). Serum bile acid concentrations were significantly elevated (14.99 ± 2.80 µM; 11.9-fold increase), accompanied by a 5.6-fold increase in BALF BA concentrations. EGCG treatment reduced serum bile acid concentrations to 9.68 ± 2.18 µM and BALF bile acid levels to 0.274 ± 0.125 µM, while decreasing *Sftpd* expression (1.29 ± 0.25-fold), sRAGE (392 ± 234 pg/mL), alveolar epithelial apoptosis (AT-1: 15.0 ± 3.8%; AT-2: 22.1 ± 7.0%), and MDA levels (0.36 ± 0.10 nmol/mg tissue). In parallel, EGCG significantly restored pulmonary SOD protein levels (17.80 ± 5.91 ng/mg tissue). Mechanistically, EGCG restored the transcriptional expression of the pulmonary Keap1-Nrf2-HO-1 antioxidant pathway by suppressing *Keap1* while increasing *Nfe2l2* and *Hmox1* expression, consistent with improved endogenous antioxidant defenses and redox homeostasis. In conclusion, MASH is accompanied by pulmonary injury, alveolar epithelial apoptosis and oxidative stress, occurring in parallel with systemic and alveolar bile acid accumulation. EGCG attenuated these changes and was associated with recovery of pulmonary Keap1-Nrf2-HO-1 signaling.

## 1. Introduction

Metabolic dysfunction-associated steatotic liver disease (MASLD) is a liver condition characterized by the presence of hepatic steatosis (fat accumulation in the liver) in conjunction with at least one cardiometabolic risk factor, such as obesity, type 2 diabetes, or metabolic abnormalities, including high blood pressure, elevated triglycerides, or low HDL cholesterol. Metabolic dysfunction-associated steatohepatitis (MASH) is a progressive form of MASLD characterized by hepatic steatosis, inflammation, and fibrosis, often occurring in the context of obesity and insulin resistance [1,2,3,4,5]. While MASH has been widely studied for its hepatic complications, emerging evidence suggests that it is a systemic disorder with significant extrahepatic manifestations, including cardiovascular, renal, and pulmonary involvement [6,7,8,9,10]. Recent studies indicate a strong association between metabolic dysfunction and lung diseases [10,11]; however, the mechanistic link between MASH and pulmonary dysfunction remains incompletely understood.

Acute lung injury (ALI) and its severe form, acute respiratory distress syndrome (ARDS), are life-threatening conditions characterized by epithelial barrier disruption, oxidative stress, and an excessive inflammatory response [12,13,14]. While ALI is traditionally triggered by infectious [15,16] or toxic insults [17,18], metabolic disorders such as obesity and diabetes [6,7,8,9,10,11] have been shown to exacerbate pulmonary inflammation and impair alveolar homeostasis. Given that MASH is a chronic inflammatory and metabolic disease, it has been implicated in lung epithelial dysfunction through mechanisms including increased oxidative stress, surfactant dysregulation, and alveolar epithelial cell apoptosis [11]. These processes mirror the pathophysiological changes observed in ALI, suggesting that MASH may independently contribute to lung injury, even in the absence of acute inflammatory triggers.

One of the emerging pathways linking MASH to pulmonary dysfunction is bile acid (BA) dysregulation [19,20]. Beyond their traditional role in lipid metabolism and enterohepatic circulation [21,22], BAs have increasingly been recognized as mediators of systemic inflammatory responses [23] and lung injury [24].

We hypothesize that hepatic bile acid metabolism is disrupted in MASH, resulting in altered bile acid composition, systemic accumulation, and potential translocation to the lungs. Elevated BA levels in bronchoalveolar lavage fluid (BALF) of MASH models suggest that BA infiltration into the lungs contributes to alveolar epithelial damage and oxidative stress, further exacerbating pulmonary dysfunction. This liver–lung axis represents a novel paradigm in the progression of metabolic diseases, warranting further investigation into its role in lung pathology.

## 2. Results

### 2.1. The MASH Mouse Model Exhibits Bile Acid Accumulation in Serum and BALF

Given the metabolic nature of MASH, we hypothesized that BA, key metabolites dysregulated in liver injury, might modulate lung tissue integrity. Therefore, as an initial step, we measured total BA levels in the serum of the MASH mouse model to determine their association with liver injury. Moreover, BA levels were also assessed in BALF samples as an indicator of lung injury. Figure 1a shows that serum BA concentrations in the MASH mouse model were markedly elevated to 14.99 ± 2.80 µM compared with littermate controls, representing an 11.9-fold increase (*p* < 0.0001). In parallel, BA levels in BALF samples showed a 5.6-fold increase compared to littermate controls, indicating similar increases in BA in both liver and lung in the MASH model (Figure 1b). To determine whether elevated BA levels contribute to the pathological changes observed in the MASH model, we used epigallocatechin-3-gallate (EGCG) as a proof-of-concept intervention. EGCG is a known modulator of bile acid metabolism that acts by inhibiting bile acid synthesis in the liver, reducing intestinal absorption, and enhancing hepatic clearance [25,26,27,28,29,30]. Figure 1a shows that EGCG treatment inhibited the accumulation of BA in the serum, maintaining concentrations at 9.68 ± 2.18 µM, significantly lower than in untreated MASH mice (*p* < 0.0001), although concentrations remained significantly higher than in littermate controls (1.26 ± 0.48 µM; *p* < 0.0001). Moreover, EGCG treatment showed an inhibitory effect on BALF BA accumulation in MASH mice, with a concentration of 0.274 ± 0.125 µM no longer significantly different from that observed in littermate naïve mice (0.086 ± 0.059 µM; *p* = 0.0665; Figure 1b). Our findings collectively demonstrate that BA accumulation is a distinctive feature of the MASH model, which is closely associated with BA accumulation in the lung and allowed us to assess markers of lung injury.

### 2.2. Epigallocatechin-3-Gallate Mitigates Liver Fibrosis and Lung Injury in the MASH Mouse Model

To further characterize the effects of EGCG at the phenotypic and molecular levels in liver and lung tissue, histological sections were obtained as described in the Section 4. Figure 2a shows the effects of EGCG on liver samples, and H&E staining reveals normal hepatic architecture in littermate mice, with well-organized hepatocytes, intact sinusoids, and no steatosis or inflammation. *Ob*/*Ob*^HFD^ MASH mice exhibited hallmark features of progressive liver disease, including severe macrovesicular steatosis, hepatocyte ballooning, and inflammatory infiltrates, indicative of chronic metabolic stress. Treatment with EGCG led to a marked reduction in hepatic steatosis, accompanied by decreased hepatocyte ballooning and diminished inflammatory cell infiltration, suggesting a protective role for EGCG in attenuating MASH-associated liver injury. Littermate mice receiving EGCG showed histopathological findings similar to those of their naïve counterparts. Hepatocyte apoptosis is a key mechanism driving liver injury in MASH. Measuring apoptotic cell death directly assesses the extent of hepatocellular damage [31,32]. As shown in Figure 2b, MASH mice exhibited a significant increase in hepatocyte apoptosis (41.6 ± 13.8%) compared to littermate controls (11.7 ± 5.8%, *p* < 0.0001). Treatment with EGCG inhibited the increase in the rate of hepatocyte apoptosis in MASH mice, maintaining a similar pattern to that of naïve mice at 19.3 ± 7.8%, indicating its protective effect on hepatocyte integrity. Importantly, no significant differences in apoptosis were observed between littermate naïve mice treated with EGCG and those not treated. Moreover, the liver injury markers ALT and AST were markedly reduced following EGCG treatment. Serum ALT rose from 22.96 ± 1.22 IU/L in littermate controls to 150.60 ± 33.09 IU/L in MASH mice (*p* < 0.0001) and decreased to 43.10 ± 24.75 IU/L after EGCG administration (*p* < 0.0001 versus untreated MASH mice), a level not significantly different from littermate controls (*p* = 0.18). Serum AST rose from 29.80 ± 3.49 IU/L to 91.50 ± 33.04 IU/L in MASH mice (*p* < 0.0001) and fell to 40.00 ± 20.86 IU/L following EGCG treatment (*p* < 0.0001 versus untreated MASH mice), likewise comparable to littermate controls (*p* = 0.71) (Figure 2c,d). Furthermore, the fibrogenic genes *Acta2* (α-SMA), *Col1a1* (collagen type I), and *Tgfb1* (transforming growth factor-β) were quantified by RT-PCR. As shown in Figure 2e, *Acta2* expression was significantly elevated in MASH mice (2.57 ± 0.71-fold) compared to littermate controls (1.04 ± 0.14-fold; *p* < 0.0001). Notably, EGCG treatment reduced *Acta2* expression to 1.10 ± 0.41-fold (*p* < 0.0001 versus untreated MASH), a level not significantly different from littermate controls (*p* > 0.05). *Col1a1* and *Tgfb1* changed in the same direction but were only partially corrected. *Col1a1* expression rose from 1.07 ± 0.22-fold in littermate controls to 2.71 ± 0.60-fold in MASH mice (*p* < 0.0001) and fell to 1.53 ± 0.37-fold following EGCG treatment (*p* < 0.0001 versus untreated MASH mice), while *Tgfb1* rose from 1.13 ± 0.11-fold to 5.38 ± 1.40-fold (*p* < 0.0001) and fell to 2.85 ± 1.42-fold (*p* < 0.0001 versus untreated MASH mice) (Figure 2f,g). In contrast to *Acta2*, however, both *Col1a1* (*p* = 0.0288) and *Tgfb1* (*p* = 0.0013) remained significantly elevated relative to littermate controls after treatment, indicating partial rather than complete resolution of the fibrogenic transcriptional program. No significant differences in fibrogenic gene expression were observed between EGCG-treated and untreated littermate controls.

We next examined whether EGCG, which normalized hepatic injury and reduced systemic BA levels, was also associated with preservation of pulmonary architecture. H&E and Sirius Red staining were performed to evaluate pulmonary histopathological alterations and collagen deposition (Figure 3a). Lung sections from littermate control mice exhibited preserved alveolar architecture with thin alveolar septa and well-aerated alveolar spaces. Only minimal focal peribronchiolar inflammatory cell infiltration was observed, consistent with normal pulmonary histology. Similarly, lungs from littermate mice treated with EGCG displayed a nearly normal histological appearance, with intact alveolar architecture, minimal inflammatory infiltrates, and no evidence of tissue remodeling, indicating that EGCG did not alter normal lung morphology. In contrast, lung tissue from *Ob*/*Ob*^HFD^ MASH mice demonstrated mild-to-moderate histopathological alterations characterized by increased peribronchiolar and interstitial inflammatory cell infiltration, focal thickening of the alveolar septa, and partial distortion of the normal alveolar architecture. Although the overall pulmonary structure remained preserved, these changes were consistent with inflammatory lung injury associated with obesity-related metabolic dysfunction. Treatment with EGCG partially ameliorated these histopathological abnormalities. Lung sections from *Ob*/*Ob*^HFD^ MASH mice receiving EGCG showed reduced inflammatory cell infiltration, less prominent septal thickening, and improved preservation of alveolar architecture compared with untreated *Ob*/*Ob*^HFD^ MASH mice. Although mild inflammatory changes persisted, the overall tissue morphology appeared less affected, suggesting that EGCG attenuated obesity-associated pulmonary injury.

Sirius Red staining was performed to assess collagen deposition and early fibrotic remodeling (Figure 3a). Lung sections from littermate control mice exhibited minimal collagen deposition, limited to the normal perivascular, peribronchiolar, and pleural connective tissue, without evidence of interstitial fibrosis. A similar staining pattern was observed in littermate mice treated with EGCG, confirming that EGCG administration did not induce collagen accumulation in healthy lungs. In *Ob*/*Ob*^HFD^ MASH mice, Sirius Red staining demonstrated increased collagen deposition predominantly surrounding bronchioles and blood vessels, with mild extension into the adjacent interstitial tissue, consistent with early extracellular matrix remodeling. In contrast, EGCG-treated *Ob*/*Ob*^HFD^ MASH mice exhibited reduced Sirius Red-positive staining, with decreased peribronchiolar and interstitial collagen deposition compared with untreated *Ob*/*Ob*^HFD^ MASH mice, indicating attenuation of early fibrotic remodeling. These findings indicate that EGCG attenuates early extracellular matrix remodeling in the MASH lung. Because collagen deposition was assessed descriptively rather than by morphometric quantification, these observations should be regarded as qualitative.

Pulmonary *Sftpd* mRNA expression, a marker of alveolar epithelial injury, was significantly increased in *Ob*/*Ob*^HFD^ mice and was markedly reduced following EGCG treatment, approaching levels observed in littermate controls (Figure 3b). Figure 3c shows reduced sRAGE levels in BALF samples, from 986 ± 144 pg/mL in MASH mice to 392 ± 234 pg/mL in EGCG-treated counterparts (*p* < 0.0001), reflecting improved alveolar integrity. No significant changes in sRAGE levels were observed between littermate control groups with or without EGCG treatment. Apoptosis in AT-1 and AT-2 cells was assessed to further evaluate epithelial injury (Figure 3d,e). Littermate controls exhibited basal apoptotic rates of 11.9 ± 2.7% in AT-1 and 11.1 ± 3.0% in AT-2 cells, which were unaffected by EGCG administration (*p* > 0.05 for both). In *Ob*/*Ob*^HFD^ mice, apoptosis increased to 30.0 ± 8.0% in AT-1 and 32.4 ± 7.8% in AT-2 cells (*p* < 0.0001 versus littermate controls for both cell types). EGCG treatment reduced AT-1 apoptosis to 15.0 ± 3.8% (*p* < 0.001 versus untreated MASH mice), a level not significantly different from littermate controls (*p* = 0.51). The effect on AT-2 cells was more limited: apoptosis decreased to 22.1 ± 7.0% (*p* < 0.001 versus untreated MASH mice) but remained significantly higher than in littermate controls (*p* < 0.001). EGCG therefore normalized AT-1 apoptosis while conferring only partial protection against AT-2 apoptosis.

To determine whether pulmonary injury in *Ob*/*Ob*^HFD^ mice is associated with oxidative stress, lipid peroxidation, and antioxidant status, pulmonary MDA and SOD protein levels were assessed. As shown in Figure 3f,g, *Ob*/*Ob*^HFD^ mice exhibited a marked increase in pulmonary MDA levels (0.67 ± 0.11 nmol/mg tissue) compared with littermate mice receiving PBS (0.15 ± 0.07 nmol/mg tissue, *p* < 0.001), indicating enhanced lipid peroxidation and oxidative damage. EGCG treatment significantly reduced pulmonary MDA levels in *Ob*/*Ob*^HFD^ mice to 0.36 ± 0.10 nmol/mg tissue (*p* < 0.01 versus *Ob*/*Ob*^HFD^ with PBS), approaching the levels observed in littermate controls. No significant difference in MDA levels was observed between littermate mice treated with PBS (0.15 ± 0.07 nmol/mg tissue) or EGCG (0.32 ± 0.45 nmol/mg tissue). Consistent with these findings, pulmonary SOD protein levels were markedly reduced in *Ob*/*Ob*^HFD^ mice (6.25 ± 3.30 ng/mg tissue) compared with littermate mice receiving PBS (25.30 ± 9.98 ng/mg tissue, *p* < 0.001), demonstrating a substantial impairment of endogenous antioxidant defenses. EGCG treatment significantly increased pulmonary SOD protein levels to 17.80 ± 5.91 ng/mg tissue (*p* < 0.01 versus *Ob*/*Ob*^HFD^ with PBS), although values remained lower than those observed in healthy littermate controls, indicating partial restoration of antioxidant capacity. Importantly, EGCG administration did not significantly alter SOD protein levels in littermate mice (22.75 ± 7.33 versus 25.30 ± 9.98 ng/mg tissue, *p* > 0.05).

Collectively, these findings demonstrate that MASH is accompanied by pulmonary injury, characterized by alveolar epithelial apoptosis, elevated epithelial injury biomarkers, and a marked oxidative imbalance, occurring in parallel with hepatic steatohepatitis and systemic bile acid accumulation. EGCG treatment attenuated both compartments concurrently: it normalized circulating and BALF bile acid levels and reduced hepatic injury and fibrogenic gene expression, while in the lung it decreased alveolar epithelial apoptosis, lowered Sftpd and BALF sRAGE, limited lipid peroxidation, and partially restored SOD protein levels. The coordinated improvement of hepatic and pulmonary endpoints is consistent with bile acids serving as a circulating link between the two organs. Given that the pulmonary phenotype was dominated by oxidative imbalance, we next examined whether the endogenous Keap1-Nrf2-HO-1 antioxidant axis is impaired in the MASH lung and whether it is restored by EGCG.

### 2.3. EGCG Restores Endogenous Antioxidant Defenses in the Lung via Modulation of the Keap1-Nrf2-HO-1 Signaling Pathway

To investigate whether the protective effects of EGCG against MASH-associated pulmonary injury involve modulation of endogenous antioxidant defenses, we examined the pulmonary expression of the Keap1-Nrf2-HO-1 signaling pathway, a central regulator of cellular redox homeostasis. Under physiological conditions, Keap1 binds Nrf2 in the cytoplasm and targets it for proteasomal degradation, thereby maintaining low basal antioxidant expression. During oxidative stress, dissociation of Nrf2 from Keap1 permits its stabilization and translocation to the nucleus, where it induces transcription of antioxidant and cytoprotective genes, including *Hmox1* (HO-1). Given the marked increase in pulmonary oxidative stress observed in *Ob*/*Ob*^HFD^ mice, as evidenced by elevated MDA levels, reduced SOD expression, and increased epithelial apoptosis, we hypothesized that chronic metabolic stress impairs the pulmonary Keap1-Nrf2-HO-1 axis and that EGCG restores this antioxidant pathway.

As shown in Figure 4a, *Ob*/*Ob*^HFD^ mice exhibited a significant increase in pulmonary *Keap1* expression (1.87 ± 0.32-fold) compared with littermate controls (1.02 ± 0.11-fold), suggesting enhanced repression of endogenous antioxidant responses. This increase was accompanied by a marked suppression of *Nfe2l2* expression (0.54 ± 0.14-fold versus 1.04 ± 0.15-fold in littermate controls; Figure 4b), indicating impairment of the major cellular antioxidant regulator. Consistent with reduced Nrf2 signaling, expression of the downstream antioxidant enzyme *Hmox1* was also significantly decreased in *Ob*/*Ob*^HFD^ mice (0.744 ± 0.050-fold versus 1.06 ± 0.13-fold in controls; Figure 4c). Collectively, these findings demonstrate that obesity-associated MASH is accompanied by disruption of pulmonary antioxidant defenses and impairment of the Keap1-Nrf2-HO-1 signaling axis.

Importantly, EGCG treatment significantly attenuated these alterations. Pulmonary *Keap1* expression decreased to 1.26 ± 0.24-fold following EGCG administration (Figure 4a), whereas *Nfe2l2* expression increased to 1.28 ± 0.19-fold (Figure 4b) and *Hmox1* expression was restored to 1.32 ± 0.29-fold (Figure 4c), approaching or modestly exceeding levels observed in healthy littermate mice. Notably, EGCG treatment had minimal effects on the expression of these genes in control animals (*Keap1*, 0.98 ± 0.23-fold; *Nfe2l2*, 0.86 ± 0.11-fold; *Hmox1*, 1.09 ± 0.11-fold; *p* > 0.05 versus vehicle-treated littermates for all three), indicating that its regulatory effects occur predominantly under conditions of metabolic and oxidative stress rather than under physiological conditions.

The restoration of the Keap1-Nrf2-HO-1 pathway by EGCG closely paralleled the reduction in lipid peroxidation, recovery of antioxidant enzyme expression, and attenuation of alveolar epithelial apoptosis observed in treated *Ob*/*Ob*^HFD^ mice. These findings suggest that EGCG exerts its pulmonary protective effects, at least in part, by normalizing endogenous antioxidant signaling and restoring redox homeostasis. Although Nrf2 expression is predominantly regulated at the post-translational level through protein stabilization and nuclear translocation, the coordinated transcriptional changes observed in *Keap1*, *Nfe2l2*, and *Hmox1* strongly support activation of this antioxidant pathway in response to EGCG treatment. Taken together, our findings identify impairment of the pulmonary Keap1-Nrf2-HO-1 axis as a previously unrecognized component of MASH-associated lung injury and suggest that reactivation of this pathway contributes substantially to the therapeutic effects of EGCG within the liver–lung axis of metabolic disease.

## 3. Discussion

Metabolic dysfunction-associated steatohepatitis (MASH) is increasingly recognized as a systemic disease with multiple extrahepatic manifestations; however, the molecular mechanisms linking hepatic metabolic injury to pulmonary dysfunction remain poorly understood. In the present study, we demonstrate that MASH promotes pulmonary injury through a previously underappreciated liver–lung axis driven by bile acid accumulation and oxidative stress. More importantly, we identify impairment of the pulmonary Keap1-Nrf2-HO-1 antioxidant signaling pathway as a novel mechanistic event underlying MASH-associated lung injury and show that EGCG effectively restores this pathway, thereby attenuating epithelial injury and oxidative damage. To our knowledge, this is the first study demonstrating that dysregulation of pulmonary Keap1-Nrf2-HO-1 signaling contributes to lung injury secondary to MASH.

A principal finding of our study is the marked accumulation of BA in both the systemic circulation and BALF of *Ob*/*Ob*^HFD^ mice. Although alterations in BA metabolism are well-established features of MASH, our findings further suggest that BA accumulation extends beyond the liver and contributes directly to pulmonary pathology. This observation supports the emerging concept of the liver–lung axis, whereby hepatic metabolic dysfunction influences distant organs through circulating metabolites and inflammatory mediators [33]. Recent studies have shown that elevated BA disrupt epithelial barrier integrity, induce mitochondrial dysfunction, activate inflammatory signaling pathways, and promote apoptosis in several epithelial tissues [34,35,36,37]. Likewise, experimental evidence demonstrates that aspiration or systemic exposure to BA induces alveolar epithelial injury, oxidative stress, surfactant dysfunction, and pulmonary inflammation [24]. Our findings extend these observations by demonstrating that chronic metabolic liver injury alone, even in the absence of a primary pulmonary insult, results in BA accumulation within the lung accompanied by alveolar epithelial apoptosis and histological evidence of lung injury.

Beyond their classical role in lipid digestion, BAs function as potent signaling molecules through receptors including farnesoid X receptor (FXR) and Takeda G protein-coupled receptor 5 (TGR5), both of which are expressed in pulmonary tissues [38]. Dysregulated BA signaling has been implicated in epithelial barrier disruption, immune activation, mitochondrial dysfunction, and excessive ROS production, thereby promoting inflammatory tissue injury [23,24,38,39,40]. Consistent with these reports, MASH mice in our study exhibited elevated pulmonary lipid peroxidation, depletion of endogenous antioxidant defenses, increased surfactant protein D and sRAGE levels, and extensive apoptosis of both alveolar type I and type II epithelial cells. Collectively, these findings support the concept that BA-mediated oxidative stress represents a central mechanism linking hepatic dysfunction to pulmonary injury.

The most critical mechanistic finding of this study is the identification of impaired pulmonary Keap1-Nrf2-HO-1 signaling as a key contributor to MASH-associated lung injury. The Keap1-Nrf2 pathway is widely recognized as the master regulator of cellular antioxidant defense. Under physiological conditions, Keap1 sequesters Nrf2 within the cytoplasm and promotes its ubiquitination and proteasomal degradation. During oxidative stress, conformational modification of Keap1 permits stabilization and nuclear translocation of Nrf2, where it transcriptionally activates numerous cytoprotective genes including heme oxygenase-1 (HO-1), NAD(*p*)H quinone oxidoreductase-1 (NQO1), glutamate-cysteine ligase, glutathione peroxidases, and superoxide-detoxifying enzymes that collectively restore intracellular redox homeostasis [41,42,43,44,45]. Numerous experimental studies have demonstrated that activation of Nrf2 protects against lipopolysaccharide-induced acute lung injury, hyperoxia, ventilator-induced lung injury, pulmonary fibrosis, and chronic obstructive pulmonary disease by suppressing oxidative stress, inflammatory cytokine production, mitochondrial dysfunction, and epithelial apoptosis [42,43,44,45]. Conversely, genetic deletion or pharmacological inhibition of Nrf2 markedly exacerbates pulmonary oxidative injury and inflammatory responses, highlighting its indispensable role in maintaining lung homeostasis.

Despite extensive evidence supporting the protective role of Nrf2 in pulmonary diseases, its involvement in metabolic liver disease-associated lung injury has remained largely unexplored. Our study fills this important knowledge gap by demonstrating that *Ob*/*Ob*^HFD^ mice exhibit increased pulmonary Keap1 expression accompanied by marked suppression of Nrf2 and HO-1 expression. These molecular alterations closely paralleled increased MDA concentrations, depletion of SOD, and enhanced apoptosis of alveolar epithelial cells, indicating profound impairment of endogenous antioxidant defenses. These findings suggest that chronic metabolic stress associated with MASH suppresses pulmonary antioxidant signaling, thereby rendering the lung more susceptible to oxidative injury. Thus, impairment of the Keap1-Nrf2-HO-1 pathway appears to represent a previously unrecognized molecular mechanism linking hepatic metabolic dysfunction with pulmonary epithelial damage.

Importantly, EGCG effectively reversed these molecular abnormalities by suppressing pulmonary Keap1 expression while restoring both Nrf2 and HO-1 expression toward basal levels. Restoration of this antioxidant pathway closely paralleled reduced lipid peroxidation, recovery of SOD expression, decreased epithelial apoptosis, improved alveolar integrity, and attenuation of histological lung injury. These observations strongly suggest that activation of endogenous antioxidant signaling represents a major downstream mechanism responsible for the pulmonary protective effects of EGCG. Although our analyses primarily evaluated transcriptional changes, the coordinated regulation of Keap1, Nrf2, and HO-1 together with the accompanying functional improvements strongly supports restoration of antioxidant homeostasis within the injured lung.

The ability of EGCG to activate the Nrf2 pathway has been reported in multiple experimental models of liver, kidney, cardiovascular, and pulmonary diseases. EGCG interacts with cysteine residues within Keap1, thereby disrupting the Keap1-Nrf2 complex and facilitating Nrf2 stabilization and nuclear translocation, ultimately inducing transcription of antioxidant and cytoprotective genes [28,29,30,41,42,43,44,45]. In addition to its antioxidant activity, EGCG suppresses NF-κB activation, inhibits inflammatory cytokine production, preserves mitochondrial integrity, and attenuates tissue fibrosis. Previous studies have also demonstrated that EGCG improves MASH through modulation of gut microbiota, inhibition of bile acid synthesis, enhancement of bile acid clearance, and attenuation of hepatic oxidative stress [25,26,27,28,29,30]. Our findings substantially extend these observations by demonstrating that correction of hepatic BA dysregulation is accompanied by restoration of pulmonary Keap1-Nrf2-HO-1 signaling, thereby providing mechanistic evidence that antioxidant pathway activation links improvement of liver metabolism to protection of distant pulmonary tissues.

Taken together, our data support a sequential pathogenic model in which hepatic metabolic dysfunction promotes systemic BA accumulation, facilitating BA deposition within the lung and subsequent oxidative stress, epithelial apoptosis, and inflammatory injury. Suppression of the pulmonary Keap1-Nrf2-HO-1 pathway amplifies these pathological events by limiting endogenous antioxidant capacity, whereas EGCG interrupts this cascade through dual mechanisms: normalization of BA homeostasis and restoration of pulmonary antioxidant signaling. This integrated model provides a mechanistic explanation for liver–lung communication during MASH progression and identifies pulmonary Keap1-Nrf2-HO-1 signaling as a previously unrecognized therapeutic target.

Several limitations should be acknowledged. We quantified transcriptional expression of Keap1, Nrf2, and HO-1 but did not evaluate Nrf2 nuclear translocation or protein abundance, which would provide additional mechanistic confirmation of pathway activation. Furthermore, although our findings strongly support BA-mediated pulmonary injury, we did not investigate individual BA species or receptor-specific signaling pathways. Future studies employing Nrf2-deficient mice, pharmacological Nrf2 inhibitors, or targeted modulation of FXR and TGR5 signaling will be valuable for establishing causal relationships between BA signaling and pulmonary antioxidant responses.

In conclusion, our study demonstrates that MASH induces pulmonary injury through a liver–lung axis characterized by BA accumulation, oxidative stress, and alveolar epithelial apoptosis. More importantly, we identify impairment of the pulmonary Keap1-Nrf2-HO-1 antioxidant pathway as a previously unrecognized mechanism driving this process. Restoration of this pathway by EGCG, together with normalization of BA homeostasis, markedly attenuates pulmonary injury. These findings not only expand our understanding of the systemic consequences of MASH but also identify activation of the pulmonary Keap1-Nrf2-HO-1 axis as a promising therapeutic strategy for preventing metabolic lung injury associated with chronic liver disease.

## 4. Materials and Methods

### 4.1. Mouse Models and Study Design

Twelve-week-old male leptin-deficient B6.Cg-Lep^ob^/J (*Ob*/*Ob*) mice and age- and sex-matched wild-type (+/+) littermates were used in this study, in accordance with Hebrew University ethical regulations and NIH guidelines. *Ob*/*Ob* mice were fed a high-fat diet (Cat. no. TD.06414 [60% fat]; 18.3% kcal protein, 21.4% kcal carbohydrate, 60.3% kcal fat) for 4 weeks to generate a MASH model, whereas wild-type littermate controls received standard rodent chow for the same period. Mice of each genotype were randomly allocated using a computer-generated randomization sequence to receive either EGCG (Merck, Cat. no. E4143, Israel) dissolved in PBS, administered by oral gavage once daily at 200 mg/kg/day for 2 weeks beginning at week 2 of the feeding period, or an equivalent volume of PBS as vehicle control. Four experimental groups were therefore studied: wild-type/chow + PBS, wild-type/chow + EGCG, *Ob*/*Ob*^HFD^ + PBS, and *Ob*/*Ob*^HFD^ + EGCG (n = 10 per group, except where stated otherwise in the figure legends). All procedures were performed during the light phase between 09:00 and 12:00, and animals were fasted for 16 h before sacrifice; both conditions were held constant across groups because murine bile acid concentrations vary with circadian phase and feeding status. Histological sections were coded and evaluated by a pathologist blinded to group allocation, and all outcome measurements were performed by investigators blinded to treatment assignment. The study was designed, conducted, and reported in accordance with the ARRIVE 2.0 guidelines. On the day of sacrifice, mice were weighed and anesthetized intramuscularly with a 0.1 mL ketamine:xylazine:acepromazine mixture (4:1:1) per 30 g of body weight, then euthanized by cervical dislocation.

### 4.2. Histological and Biochemical Assessments of Mouse Lungs and Livers

The posterior one-third of the lungs and livers were fixed in 4% formalin for 24 h at room temperature and then embedded in paraffin using an automated tissue processor. Sections (7 µm) were deparaffinized by immersion in xylene. Sections were then rehydrated by passing them through a series of graded alcohols, starting with absolute alcohol and ending with distilled water. Hematoxylin and eosin (H&E) staining was used to evaluate the level of steatosis, necroinflammatory regions, and apoptotic bodies. Sirius Red F3B (0.1%) in saturated picric acid (ab150681, Abcam, Israel) was used to visualize connective tissue and collagen deposition in lungs only. Stained slides were scanned using a Zeiss microscope version 3.13. Histological image interpretation and qualitative assessment were approved by a pathologist.

### 4.3. RNA Isolation, cDNA Preparation, and Real-Time PCR

Total RNA was isolated from mouse lung and liver tissues using TRI Reagent (Bio-Lab, Cat. no. 90102331, Israel) according to the manufacturer’s instructions. RNA concentration and purity were determined using a NanoDrop ND-1000 spectrophotometer (NanoDrop Technologies, Wilmington, DE), and only samples with acceptable purity (A260/A280 ratio of 1.8–2.0) were used for downstream analyses. One microgram of total RNA was reverse-transcribed into complementary DNA (cDNA) using the High-Capacity cDNA Reverse Transcription Kit (Applied Biosystems, Israel) according to the manufacturer’s protocol. Quantitative real-time PCR (qRT-PCR) was performed using TaqMan Fast Advanced Master Mix (Applied Biosystems, Cat. no. 4371130, Israel) on a QuantStudio™ 5 Real-Time PCR System (Applied Biosystems, Cat. no. A34322, Israel). The expression of pulmonary genes, including *Sftpd* (surfactant protein D), *Keap1*, *Nfe2l2*, and *Hmox1*, as well as *Acta2* (α-SMA), *Col1a1*, and *Tgfb1*, was quantified. Gene expression was normalized to *Gapdh*, and relative expression levels were calculated using the 2^−ΔΔCt^ method, with untreated littermate mice serving as the calibrator group (fold change = 1).

### 4.4. Bronchoalveolar Lavage Fluid (BALF) Extraction from Lung Tissues

The trachea was surgically exposed and intubated with a syringe catheter, and the lungs were lavaged five times with 0.5 mL of pre-warmed PBS (2.5 mL instilled in total). The recovered fractions were pooled. The same instillation volume and lavage technique were applied to all animals. Cells were pelleted by centrifugation (500× *g*, 10 min, 4 °C), resuspended in cold PBS, counted, and immediately used for subsequent analyses. Supernatants were stored at −80 °C.

### 4.5. Bile Acids (BA) Detection in Serum and BALF

BA evaluations in serum and BALF were performed using the Trinity Biotech bile acids reagent kit (Biotest; 450A, Israel) for the enzymatic determination of BA concentration, as described by Salhab et al. [25], according to the manufacturer’s instructions.

### 4.6. Liver Injury Enzyme Assessment

Following a 16-h fasting period, whole blood was collected by cardiac puncture at the time of sacrifice and centrifuged at 5000 rpm for 30 min at 4 °C to obtain serum. Serum alanine aminotransferase (ALT; Abcam, Cat. no. ab285263, Israel) and aspartate aminotransferase (AST; Abcam, Cat. no. ab263882, Israel) activities were determined using commercially available colorimetric assay kits according to the manufacturer’s instructions. Briefly, all reagents and serum samples were equilibrated to room temperature before analysis. Standards and samples were added to a 96-well microplate, followed by the addition of the reaction mixture supplied with the kit. After incubation under the recommended conditions, absorbance was measured using a Tecan Infinite M100 microplate reader (Tecan, Switzerland). Enzyme activities were calculated from the corresponding standard curves and expressed according to the manufacturer’s recommendations.

### 4.7. Lipid Peroxidation Assay

Pulmonary lipid peroxidation was assessed by measuring malondialdehyde (MDA) levels using the Lipid Peroxidation (MDA) Assay Kit (Abcam, Cat. no. ab118970, Israel). Lung tissues were homogenized in the manufacturer’s assay buffer and centrifuged to remove insoluble material. Supernatants were processed according to the manufacturer’s protocol. Briefly, standards and tissue lysates were mixed with the MDA reaction mixture and incubated under the recommended conditions to generate a chromogenic reaction. Absorbance was measured at 532 nm using a Tecan Infinite M100 microplate reader. MDA concentrations were calculated from a standard curve and normalized to the total protein concentration of each sample.

### 4.8. Superoxide Dismutase (SOD) Measurement

Pulmonary superoxide dismutase (SOD) levels were quantified using the Mouse Superoxide Dismutase (SOD) ELISA Kit (MyBioSource, Cat. no. MBS2707323, Israel) according to the manufacturer’s instructions. Lung tissue homogenates were prepared in ice-cold lysis buffer and centrifuged to remove cellular debris. Standards and samples (100 μL) were added to antibody-coated microplate wells and incubated for 2.5 h at room temperature with gentle shaking. Wells were washed four times with the supplied wash buffer before incubation with 100 μL of the detection antibody for 1 h, followed by incubation with 100 μL of streptavidin-HRP for 45 min at room temperature. After washing, 100 μL of TMB substrate solution was added to each well, and the wells were incubated in the dark for 30 min. The reaction was terminated by adding 50 μL of stop solution, and absorbance was measured immediately at 450 nm using a Tecan Infinite M100 microplate reader. SOD concentrations were calculated from the standard curve generated for each assay.

### 4.9. Soluble Receptor for Advanced Glycation End Products (sRAGE) Detection in BALF

sRAGE evaluations in BALF were detected and quantified using the sRAGE ELISA kit (R&D; MRG00) according to the manufacturer’s instructions.

### 4.10. Alveolar Epithelial Cells Isolation and Phenotyping

Both type I and type II alveolar epithelial cells were isolated from mouse lungs, as described by Chen et al. [46], and sorted using FACSAria III Cell Sorter (BD FACSAria). Mice were euthanized. The lungs were harvested and inflated with a collagenase-based digestion buffer to separate the alveolar epithelial cells. The lung tissue was minced and incubated in the digestion buffer at 37 °C with constant agitation to dissociate the cells. After digestion, the tissue was filtered through a 70 µm mesh to remove debris. The resulting single-cell suspension was subjected to differential centrifugation to enrich epithelial cells. To specifically identify AT-1 and AT-2 cells, we used a combination of cell-surface markers on a FACSAria III Cell Sorter (BD FACSAria). AT-1 cells were identified by anti-mouse aquaporin 5 (Abcam; Cat. no. 315855, Israel), while AT-2 cells were distinguished and sorted using anti-mouse epithelial cell adhesion molecule (EpCAM, Abcam; Cat. no. ab221552, Israel) and anti-mouse surfactant protein C antibody (SP-C, Abcam; Cat. no. 211326, Israel). The staining was confirmed by comparable staining patterns following the use of isotype IgG labeled with the relevant fluorochrome [isotype negative control]. After sorting, cells were assessed by propidium iodide (PI) staining according to the kit instructions (A35110, Invitrogen). Samples ≥ 90% PI-negative cells were considered viable. Apoptosis was evaluated using annexin V staining (A35110, Invitrogen, Israel). Early apoptotic cells were defined as annexin V^+^/PI^−^ cells, and late apoptotic cells as annexin V^+^/PI^+^ cells. All stained cells were examined using a BD LSR Fortessa Cell Analyzer (Becton Dickinson, Immunofluorometry Systems) and analyzed with FCS Express 7 Software for flow cytometry.

### 4.11. Hepatocyte (HEP) Isolation

Primary hepatocytes were isolated from murine liver tissue using a two-step collagenase perfusion method. Briefly, mice were anesthetized, and the portal vein was cannulated for in situ liver perfusion. The liver was initially perfused with a calcium-free HEPES buffer (pH 7.4) to remove blood and loosen cell junctions. This was followed by a second perfusion with collagenase type IV solution (0.05% in HEPES buffer containing 5 mM CaCl_2_) at 37 °C to enzymatically digest the extracellular matrix. Once digestion was complete, the liver was excised and gently disrupted in cold William’s E medium supplemented with 10% fetal bovine serum (FBS) and 1% penicillin–streptomycin. The resulting cell suspension was filtered through a 70 µm nylon mesh and centrifuged at 50× *g* for 5 min at 4 °C to enrich for viable hepatocytes. Cells were washed twice, and viability was assessed by trypan blue exclusion, consistently yielding greater than 85% viability. Hepatocytes were either used immediately for downstream applications or cultured on collagen-coated plates as needed.

### 4.12. Statistical Analysis

Statistical analyses were performed using GraphPad Prism version 10.0 (GraphPad Software, San Diego, CA, USA). Data are presented as mean ± standard deviation (SD), and each data point represents an independent biological replicate, with the individual animal as the unit of analysis. Normality of the distribution was assessed using the Shapiro–Wilk test, and homogeneity of variance was assessed using the Brown–Forsythe test. Data were analyzed by two-way analysis of variance (ANOVA) with genotype (wild-type littermate versus *Ob*/*Ob*^HFD^) and treatment (vehicle versus EGCG) as fixed factors, including the genotype × treatment interaction term, followed by Tukey’s multiple comparisons test for all pairwise comparisons between the four experimental groups. Adjusted *p* values are reported throughout, and a two-tailed *p* < 0.05 was considered statistically significant. The number of biological replicates (n) for each experiment is stated in the corresponding figure legend; group sizes differ between endpoints, with serum bile acid and BALF sRAGE determinations available for eight animals per group, hepatocyte apoptosis for seven, and pulmonary *Keap1*, *Nfe2l2*, and *Hmox1* expression for five, owing to limitations of sample volume and RNA yield. No formal sample size calculation was performed; group sizes were based on those commonly used in comparable murine models of metabolic liver disease.

## 5. Conclusions

MASH induces pulmonary injury through a liver–lung axis characterized by bile acid accumulation, oxidative stress, and disruption of the pulmonary Keap1-Nrf2-HO-1 antioxidant pathway. EGCG attenuated these pathological changes by restoring bile acid homeostasis and antioxidant signaling, thereby reducing both hepatic and pulmonary injury. These findings identify the pulmonary Keap1-Nrf2-HO-1 axis as a potential therapeutic target in MASH-associated lung injury.

## Figures and Tables

**Figure 1:**
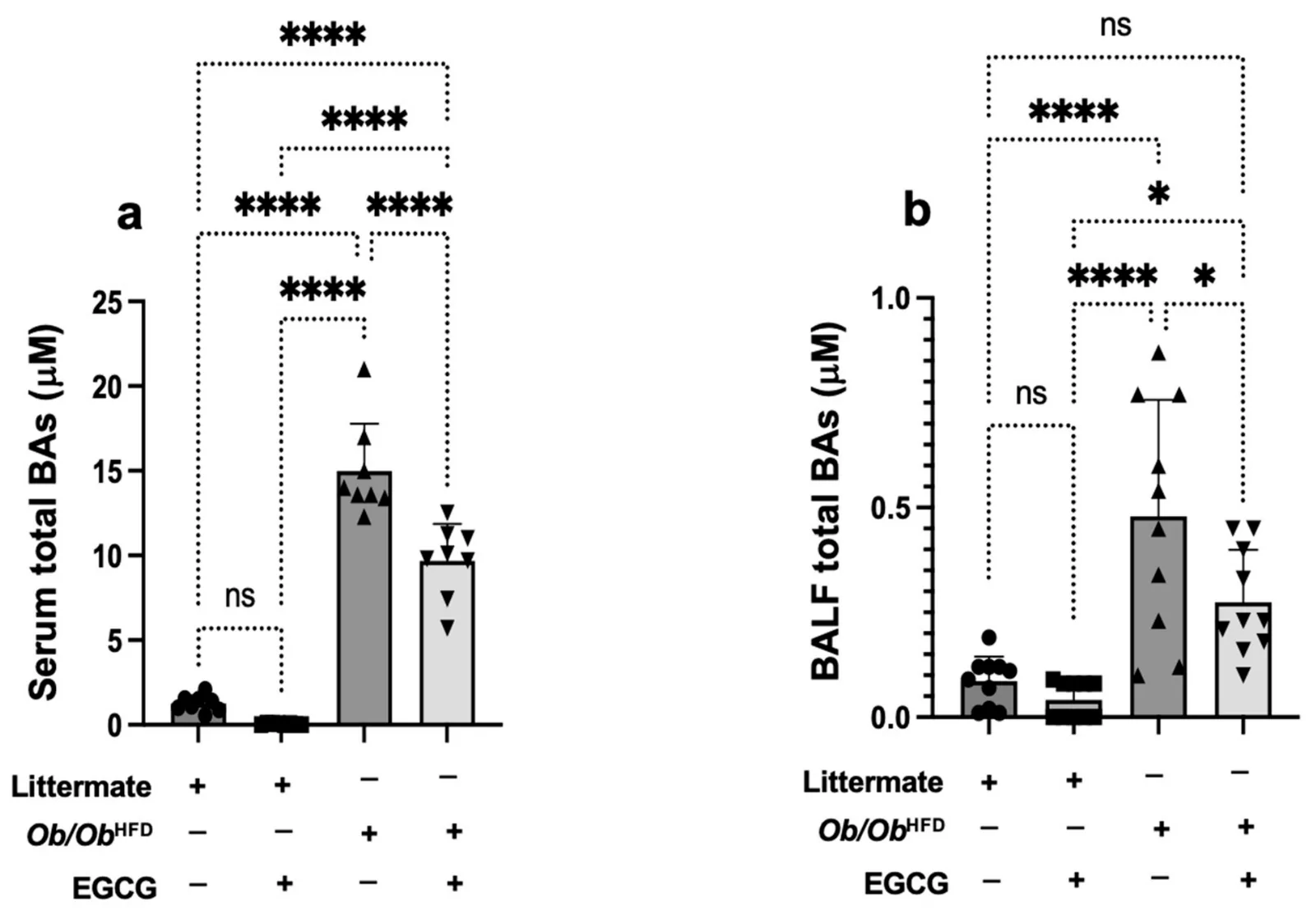
Elevated circulating and pulmonary bile acids in MASH are reduced by EGCG treatment. Total BA concentrations in (**a**) serum and (**b**) BALF were measured using an enzymatic assay in *Ob*/*Ob*^HFD^ mice, with or without treatment (serum, n = 8 per group; BALF, n = 10 per group). EGCG was administered at 200 mg/kg/day via oral gavage for two weeks, starting from week 2 of high-fat diet feeding. Control mice received an equivalent volume of PBS as a vehicle. All data are presented as mean ± SD. Statistical analysis was performed using two-way ANOVA followed by Tukey’s multiple comparisons test. Statistical significance was defined as *p* < 0.05 (* *p* < 0.05, **** *p* < 0.0001; ns, not significant).

**Figure 2:**
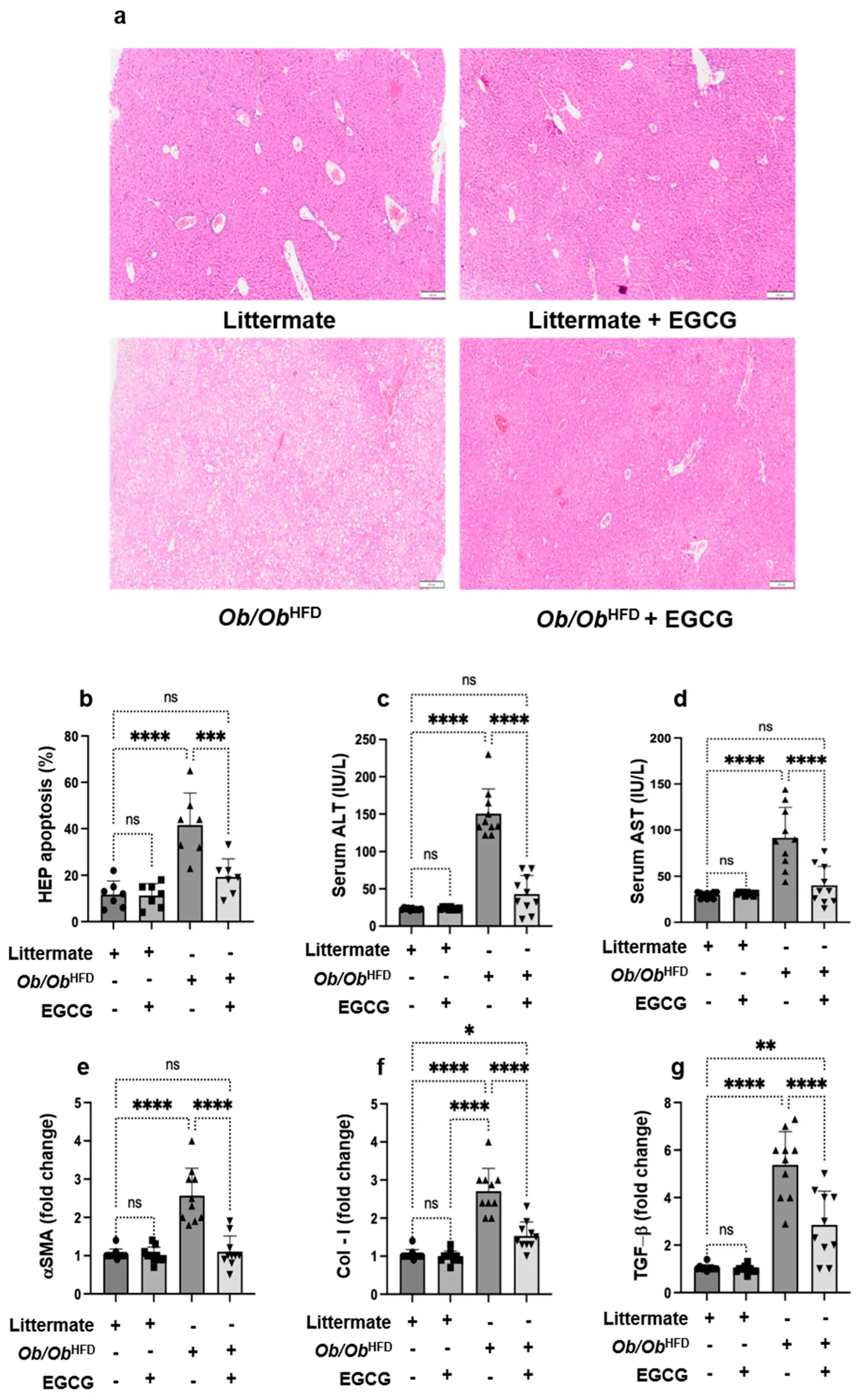
EGCG treatment attenuates liver injury, apoptosis, and fibrogenic gene expression in MASH model mice. *Ob*/*Ob*^HFD^ MASH mice treated with or without EGCG (n = 10 per group, except hepatocyte apoptosis, n = 7 per group) were evaluated for (**a**) H&E staining of random sections of the mouse livers (original magnification, ×10). (**b**) Apoptosis of hepatocytes (HEP) was analyzed by flow cytometry and expressed as the percentage of Annexin V^+^/PI^−^ cells. The expression of the liver injury markers (**c**) serum ALT and (**d**) serum AST was measured using a colorimetric assay, and that of (**e**) *Acta2*, (**f**) *Col1a1*, and (**g**) *Tgfb1* mRNA was assessed by RT-PCR. All data are presented as mean ± SD. Statistical analysis was performed using two-way ANOVA followed by Tukey’s multiple comparisons test. Statistical significance was defined as *p* < 0.05 (* *p* < 0.05, ** *p* < 0.01, *** *p* < 0.001, **** *p* < 0.0001; ns, not significant).

**Figure 3:**
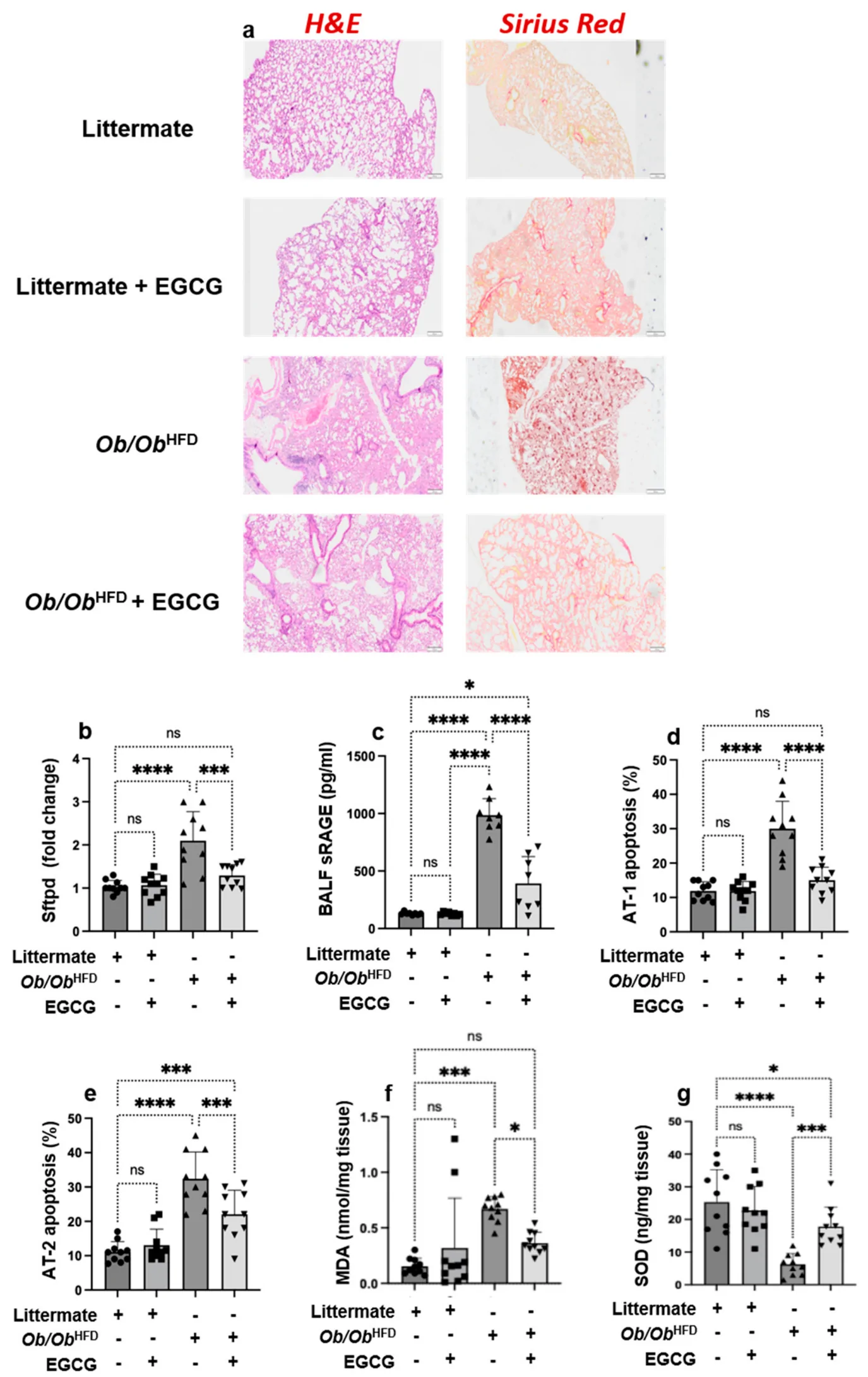
EGCG treatment attenuates lung injury in the MASH model mice. *Ob*/*Ob*^HFD^ MASH mice treated with or without EGCG (n = 10 per group, except BALF sRAGE, n = 8 per group) were evaluated for (**a**) H&E and Sirius Red staining and (**b**) mRNA expression of *Sftpd* (surfactant protein D) in lung tissue, assessed by RT-PCR. Results are shown as fold changes relative to naïve littermate controls, with *Gapdh* as the internal control. (**c**) Soluble RAGE (sRAGE) levels in bronchoalveolar lavage fluid (BALF) were measured by ELISA. (**d**,**e**) Apoptosis of alveolar epithelial cells type I (AT-1) and type II (AT-2), analyzed by flow cytometry and expressed as the percentage of Annexin V^+^/PI^−^ cells. (**f**) Malondialdehyde (MDA) levels were measured using a colorimetric assay. (**g**) Superoxide dismutase (SOD) protein levels were quantified by ELISA. All data are presented as mean ± SD. Statistical analysis was performed using two-way ANOVA followed by Tukey’s multiple comparisons test. Statistical significance was defined as *p* < 0.05 (* *p* < 0.05, *** *p* < 0.001, **** *p* < 0.0001; ns, not significant).

**Figure 4:**
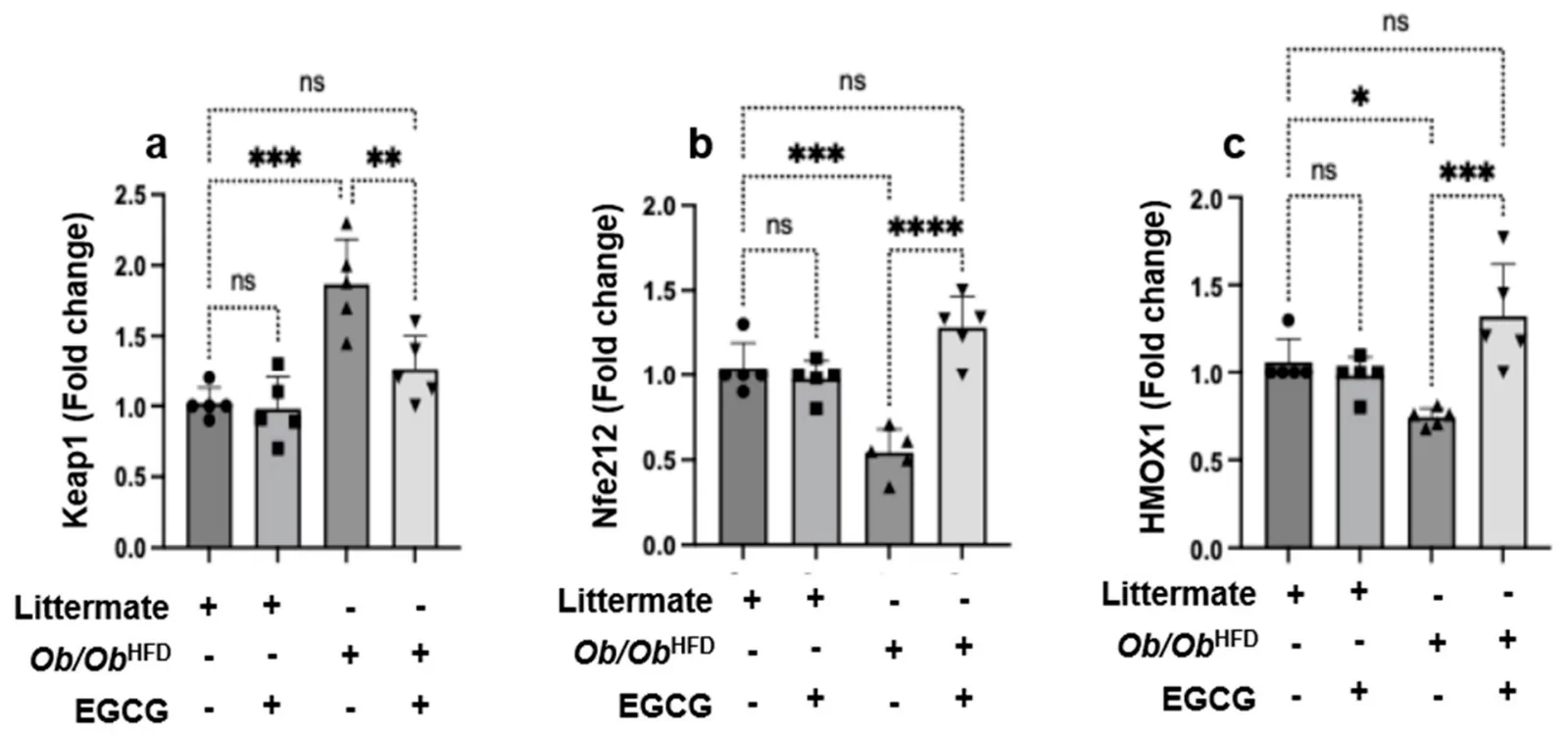
EGCG restores the pulmonary Keap1–Nrf2–HO-1 antioxidant signaling pathway in *Ob*/*Ob*^HFD^ MASH mice. Pulmonary expression of the antioxidant signaling pathway components (**a**) *Keap1*, (**b**) *Nfe2l2* (Nrf2), and (**c**) *Hmox1* (HO-1) was quantified by RT-PCR in lung tissues obtained from littermate and *Ob*/*Ob*^HFD^ mice treated with PBS or EGCG (200 mg/kg/day) for two weeks (n = 5 per group). Gene expression levels were normalized to the housekeeping gene *Gapdh* and expressed as fold change relative to littermate mice receiving PBS, which were set to 1. All data are presented as mean ± SD. Statistical analysis was performed using two-way ANOVA followed by Tukey’s multiple comparisons test. Statistical significance was defined as *p* < 0.05 (* *p* < 0.05, ** *p* < 0.01, *** *p* < 0.001, **** *p* < 0.0001; ns, not significant).

## Data Availability

The study’s original findings and contributions are detailed in the article itself. For any additional inquiries or information, we recommend contacting the study’s corresponding authors. They can provide further clarification and address any specific questions related to the research.

## Abbreviations

The following abbreviations are used in this manuscript:

ALI	Acute Lung Injury
ALT	Alanine Aminotransferase
AST	Aspartate Aminotransferase
AT-1	Alveolar Type I Cells
AT-2	Alveolar Type II Cells
BA	Bile Acid(s)
BALF	Bronchoalveolar Lavage Fluid
EGCG	Epigallocatechin-3-gallate
FXR	Farnesoid X Receptor
H&E	Hematoxylin and Eosin
HFD	High-Fat Diet
HO-1	Heme Oxygenase-1
Keap1	Kelch-like ECH-associated Protein 1
MASH	Metabolic Dysfunction-Associated Steatohepatitis
MASLD	Metabolic Dysfunction-Associated Steatotic Liver Disease
MDA	Malondialdehyde
Nrf2 (Nfe2l2)	Nuclear Factor Erythroid 2-Related Factor 2
PBS	Phosphate-Buffered Saline
qRT-PCR	Quantitative Real-Time Polymerase Chain Reaction
ROS	Reactive Oxygen Species
sRAGE	Soluble Receptor for Advanced Glycation End Products
Sftpd (SP-D)	Surfactant Protein D
SOD	Superoxide Dismutase
TGF-β	Transforming Growth Factor-β
α-SMA	Alpha-Smooth Muscle Actin

## References

[1] Huh Y., Cho Y.J., Nam G.E. (2022). Recent epidemiology and risk factors of nonalcoholic fatty liver disease. J. Obes. Metab. Syndr..

[2] Younossi Z.M., Koenig A.B., Abdelatif D., Fazel Y., Henry L., Wymer M. (2016). Global epidemiology of nonalcoholic fatty liver disease—Meta-analytic assessment of prevalence, incidence, and outcomes. Hepatology.

[3] Steinberg G.R., Valvano C.M., De Nardo W., Watt M.J. (2025). Integrative metabolism in MASLD and MASH: Pathophysiology and emerging mechanisms. J. Hepatol..

[4] Do A., Zahrawi F., Mehal W.Z. (2025). Therapeutic landscape of metabolic dysfunction-associated steatohepatitis (MASH). Nat. Rev. Drug Discov..

[5] Rinella M., Charlton M. (2016). The globalization of nonalcoholic fatty liver disease: Prevalence and impact on world health. Hepatology.

[6] Gutiérrez-Cuevas J., Santos A., Armendariz-Borunda J. (2021). Pathophysiological molecular mechanisms of obesity: A link between MAFLD and NASH with cardiovascular diseases. Int. J. Mol. Sci..

[7] Duell P.B., Welty F.K., Miller M., Chait A., Hammond G., Ahmad Z., Cohen D.E., Horton J.D., Pressman G.S., Toth P.P. (2022). Nonalcoholic fatty liver disease and cardiovascular risk: A scientific statement from the American Heart Association. Arterioscler. Thromb. Vasc. Biol..

[8] Byrne C.D., Targher G. (2015). NAFLD: A multisystem disease. J. Hepatol..

[9] Caussy C., Aubin A., Loomba R. (2021). The relationship between type 2 diabetes, NAFLD, and cardiovascular risk. Curr. Diabetes Rep..

[10] Jia Y., Li D., You Y., Yu J., Jiang W., Liu Y., Zeng R., Wan Z., Lei Y., Liao X. (2023). Multi-system diseases and death trajectory of metabolic dysfunction-associated fatty liver disease: Findings from the UK Biobank. BMC Med..

[11] Miao L., Yang L., Guo L.S., Shi Q.Q., Zhou T.F., Chen Y., Zhang H., Cai H., Xu Z.W., Yang S.Y. (2022). Metabolic dysfunction-associated fatty liver disease is associated with greater impairment of lung function than nonalcoholic fatty liver disease. J. Clin. Transl. Hepatol..

[12] Mokrá D. (2020). Acute lung injury—From pathophysiology to treatment. Physiol. Res..

[13] Fan E., Brodie D., Slutsky A.S. (2018). Acute respiratory distress syndrome: Advances in diagnosis and treatment. JAMA.

[14] Verma N., Hochhegger B., Mukhopadhyay S., Teixeira e Silva Torres P.P., Mohammed T.L. (2025). Acute lung injury. J. Thorac. Imaging.

[15] Long M.E., Mallampalli R.K., Horowitz J.C. (2022). Pathogenesis of pneumonia and acute lung injury. Clin. Sci..

[16] Kumar V. (2020). Pulmonary innate immune response determines the outcome of inflammation during pneumonia and sepsis-associated acute lung injury. Front. Immunol..

[17] Elfsmark L., Ågren L., Akfur C., Jonasson S. (2022). Ammonia exposure by intratracheal instillation causes severe and deteriorating lung injury and vascular effects in mice. Inhal. Toxicol..

[18] Elfsmark L., Ågren L., Akfur C., Bucht A., Jonasson S. (2018). 8-Isoprostane is an early biomarker for oxidative stress in chlorine-induced acute lung injury. Toxicol. Lett..

[19] Lai J., Luo L., Zhou T., Feng X., Ye J., Zhong B. (2023). Alterations in circulating bile acids in metabolic dysfunction-associated steatotic liver disease: A systematic review and meta-analysis. Biomolecules.

[20] Tang X., Zhou Y., Xia L., Lin X., Zhu Y., Chen M., Wang J., Li Y. (2025). Multifaceted interactions between bile acids, their receptors, and MASH: From molecular mechanisms to clinical therapeutics. Molecules.

[21] Molinaro A., Wahlström A., Marschall H.U. (2018). Role of bile acids in metabolic control. Trends Endocrinol. Metab..

[22] McGlone E.R., Bloom S.R. (2019). Bile acids and the metabolic syndrome. Ann. Clin. Biochem..

[23] Cai J., Sun L., Gonzalez F.J. (2022). Gut microbiota-derived bile acids in intestinal immunity, inflammation, and tumorigenesis. Cell Host Microbe.

[24] De Luca D., Alonso A., Autilio C. (2022). Bile acid-induced lung injury: Update of reverse translational biology. Am. J. Physiol. Lung Cell. Mol. Physiol..

[25] Salhab A., Amer J., Lu Y., Safadi R. (2022). Sodium+/taurocholate cotransporting polypeptide as target therapy for liver fibrosis. Gut.

[26] Zhu M.Z., Zhou F., Ouyang J., Wang Q.Y., Li Y.L., Wu J.L., Huang J.A., Liu Z.H. (2021). Combined use of epigallocatechin-3-gallate (EGCG) and caffeine in low doses exhibits marked anti-obesity synergy through regulation of gut microbiota and bile acid metabolism. Food Funct..

[27] Naito Y., Ushiroda C., Mizushima K., Inoue R., Yasukawa Z., Abe A., Takagi T. (2020). Epigallocatechin-3-gallate (EGCG) attenuates non-alcoholic fatty liver disease via modulating the interaction between gut microbiota and bile acids. J. Clin. Biochem. Nutr..

[28] He M., Chu T., Wang Z., Feng Y., Shi R., He M., Feng S., Lu L., Cai C., Fang F. (2023). Inhibition of macrophage inflammasome activation via autophagic degradation of HMGB1 by EGCG ameliorates HBV-induced liver injury and fibrosis. Front. Immunol..

[29] Tang G., Xu Y., Zhang C., Wang N., Li H., Feng Y. (2021). Green tea and epigallocatechin gallate (EGCG) for the management of nonalcoholic fatty liver diseases (NAFLD): Insights into the role of oxidative stress and antioxidant mechanism. Antioxidants.

[30] George J., Tsuchishima M., Tsutsumi M. (2022). Epigallocatechin-3-gallate inhibits osteopontin expression and prevents experimentally induced hepatic fibrosis. Biomed. Pharmacother..

[31] Qiao J.T., Cui C., Qing L., Wang L.S., He T.Y., Yan F., Liu F.Q., Shen Y.H., Hou X.G., Chen L. (2018). Activation of the STING-IRF3 pathway promotes hepatocyte inflammation, apoptosis and induces metabolic disorders in nonalcoholic fatty liver disease. Metabolism.

[32] LeFort K.R., Rungratanawanich W., Song B.J. (2024). Contributing roles of mitochondrial dysfunction and hepatocyte apoptosis in liver diseases through oxidative stress, post-translational modifications, inflammation, and intestinal barrier dysfunction. Cell. Mol. Life Sci..

[33] Young R.P., Hopkins R.J., Marsland B. (2016). The gut-liver-lung axis: Modulation of the innate immune response and its possible role in chronic obstructive pulmonary disease. Am. J. Respir. Cell Mol. Biol..

[34] Chen X., Oshima T., Tomita T., Fukui H., Watari J., Matsumoto T., Miwa H. (2011). Acidic bile salts modulate the squamous epithelial barrier function by modulating tight junction proteins. Am. J. Physiol. Gastrointest. Liver Physiol..

[35] Chen X., Oshima T., Shan J., Fukui H., Watari J., Miwa H. (2012). Bile salts disrupt human esophageal squamous epithelial barrier function by modulating tight junction proteins. Am. J. Physiol. Gastrointest. Liver Physiol..

[36] Manni M.L., Heinrich V.A., Buchan G.J., O’Brien J.P., Uvalle C., Cechova V., Koudelka A., Ukani D., Rawas-Qalaji M., Oury T.D. (2021). Nitroalkene fatty acids modulate bile acid metabolism and lung function in obese asthma. Sci. Rep..

[37] Zhao R., Liang X., Zhao M., Liu S.L., Huang Y., Idell S., Li X., Ji H.L. (2014). Correlation of apical fluid-regulating channel proteins with lung function in human COPD lungs. PLoS ONE.

[38] Fiorucci S., Biagioli M., Zampella A., Distrutti E. (2018). Bile acid-activated receptors regulate innate immunity. Front. Immunol..

[39] Perez M.J., Briz O. (2009). Bile-acid-induced cell injury and protection. World J. Gastroenterol..

[40] Shi L., Jin L., Huang W. (2023). Bile acids, intestinal barrier dysfunction, and related diseases. Cells.

[41] Bellezza I., Giambanco I., Minelli A., Donato R. (2018). Nrf2-Keap1 signaling in oxidative and reductive stress. Biochim. Biophys. Acta Mol. Cell Res..

[42] Baird L., Yamamoto M. (2020). The molecular mechanisms regulating the KEAP1-NRF2 pathway. Mol. Cell. Biol..

[43] Dodson M., de la Vega M.R., Cholanians A.B., Schmidlin C.J., Chapman E., Zhang D.D. (2019). Modulating NRF2 in disease: Timing is everything. Annu. Rev. Pharmacol. Toxicol..

[44] Cuadrado A., Rojo A.I., Wells G., Hayes J.D., Cousin S.P., Rumsey W.L., Attucks O.C., Franklin S., Levonen A.L., Kensler T.W. (2019). Therapeutic targeting of the NRF2 and KEAP1 partnership in chronic diseases. Nat. Rev. Drug Discov..

[45] Lu M.C., Ji J.A., Jiang Z.Y., You Q.D. (2016). The Keap1-Nrf2-ARE pathway as a potential preventive and therapeutic target: An update. Med. Res. Rev..

[46] Chen Q., Liu Y. (2020). Isolation and culture of mouse alveolar type II cells to study type II to type I cell differentiation. STAR Protoc..

